# Beyond the Cardio–Renal–Metabolic Axis: Emerging Therapeutic Targets and Novel Mechanisms of Action of Flozins

**DOI:** 10.3390/jcm14186348

**Published:** 2025-09-09

**Authors:** Wojciech Matuszewski, Lena Tomaszek, Michał Szklarz, Jan Marek Górny, Bernard Kordas, Joanna Rutkowska, Judyta Juranek

**Affiliations:** 1 Clinic of Endocrinology, Diabetology and Internal Medicine, School of Medicine, Collegium Medicum, University of Warmia and Mazury, 10-082 Olsztyn, Poland; michszklarz@gmail.com (M.S.); jangorny245@gmail.com (J.M.G.); rutkowskaj14@gmail.com (J.R.); 2 Department of Pharmacology and Toxicology, School of Medicine, Collegium Medicum, University of Warmia and Mazury, 10-082 Olsztyn, Poland; tomaszek.lena@gmail.com; 3 Department of Human Physiology and Pathophysiology, University of Warmia and Mazury, 10-082 Olsztyn, Poland; kordas.ber@gmail.com (B.K.); judytajuranek@gmail.com (J.J.)

**Keywords:** SGLT2 inhibitors, diabetes mellitus, pleiotropy, metabolism

## Abstract

Contemporary diabetes management is progressively moving away from a glucocentric approach, with growing expectations that novel antidiabetic agents offer benefits beyond glycaemic control. Sodium–glucose cotransporter 2 inhibitors (SGLT2i) have emerged as a cornerstone in the treatment of type 2 diabetes mellitus (T2DM). In addition to reducing blood glucose levels by promoting renal glucose excretion, these agents contribute significantly to cardio–renal–metabolic protection and are associated with improved cardiovascular outcomes and prolonged survival. Although SGLT2 inhibitors do not exhibit a class effect in all clinical aspects, growing evidence suggests their potential in a variety of additional therapeutic areas. We conducted an in-depth review of current scientific literature and clinical studies regarding this class of drugs. SGLT2 inhibitors demonstrate neuroprotective properties and may provide benefits in neurodegenerative disorders such as Alzheimer’s and Parkinson’s disease, potentially through the improvement of mitochondrial function and attenuation of inflammatory responses. Their anti-inflammatory and antioxidative effects are closely linked to reductions in cardiac and renal fibrosis. Other observed benefits include weight loss, improved insulin sensitivity, normalization of serum uric acid, and a reduction in hepatic steatosis—each with important metabolic implications. Furthermore, SGLT2 inhibitors have been shown to positively influence iron metabolism and improve erythrocyte indices. Emerging data also indicate beneficial effects in women with polycystic ovary syndrome. Another promising area of investigation involves the modulation of Klotho protein expression and support of vascular homeostasis. In oncology, SGLT2 inhibitors are gaining attention, with encouraging preclinical results observed in malignancies such as pancreatic, thyroid, breast, and lung cancers. Based on a comprehensive evaluation of the existing body of evidence, it is anticipated that the clinical indications for SGLT2 inhibitors will expand beyond the cardio–renal–metabolic axis in the near future.

## 1. Introduction

Sodium-glucose co-transporter 2 inhibitors (SGLT2i) represent a pharmacological class primarily used in the management of type 2 diabetes mellitus (T2DM). The introduction of SGLT2 inhibitors into clinical practice in 2012 marked a major milestone in diabetes therapy. Dapagliflozin was the first SGLT2 inhibitor to be approved for use, followed by other notable drugs such as canagliflozin, empagliflozin, and ertugliflozin, all of which received approval from the European Medicines Agency (EMA) [1]. In the years that followed, additional SGLT2 inhibitors were introduced, including ipragliflozin, sotagliflozin, remogliflozin etabonate, luseogliflozin, and tofogliflozin, further expanding the options available for treating T2DM [2]. In addition to their antihyperglycemic effects, these agents have demonstrated efficacy in the treatment of heart failure and chronic kidney disease (CKD) [3,4,5,6]. Discoveries regarding their extended effects have significantly shifted the perception of this drug class, leading to updates in clinical guidelines. Although the mechanism of action of SGLT2 inhibitors, reducing renal glucose reabsorption, is well established, increasing attention has been given to their additional therapeutic effects. In recent years, an increasing number of scientific reports have highlighted the pleiotropic actions of SGLT2 inhibitors, with potential benefits extending beyond conventional diabetes treatment [7]. Studies suggest that SGLT2 inhibitors exhibit neuroprotective and anti-inflammatory properties and reduce serum uric acid levels. These emerging effects open up new possibilities for the treatment and prevention of such diseases as Alzheimer’s Disease (AD), Parkinson’s Disease (PD) or Metabolic Dysfunction-Associated Steatotic Liver Disease (MASLD), as well as their potential complications. What sets this review apart from prior publications is its comprehensive focus on the broad spectrum of pleiotropic effects of SGLT2 inhibitors and their potential repurposing for diverse therapeutic applications beyond diabetes and cardiovascular diseases. While previous reviews have mainly concentrated on glycemic control and heart or kidney outcomes, this article aims to synthesize the latest evidence on neuroprotection, metabolic regulation, inflammation modulation, and other emerging areas, thereby providing a more holistic overview of the evolving role of SGLT2 inhibitors in modern medicine. The aim of this review is to present the current state of knowledge regarding the pleiotropic effects of SGLT2 inhibitors and to discuss their potential applications beyond diabetes treatment. Repurposing these agents for novel therapeutic indications could mark a significant advancement in the treatment and prevention of various diseases, especially in individuals with metabolic syndrome.

## 2. Materials and Methods

The study presents an analysis of current literature data on the use of SGLT2 inhibitors, with particular emphasis on their pleiotropic effects. It also discusses newly identified mechanisms of action and potential future therapeutic targets for this drug class. A Generative AI tool (ChatGPT, OpenAI) was used to assist with translation of technical content, organization of text, and formatting of citations. All scientific content was verified and edited by the authors to ensure accuracy and integrity.

## 3. Results and Discussion

### 3.1. Mechanism of Action and Therapeutic Effects of SGLT-2i

**Figure 1:**
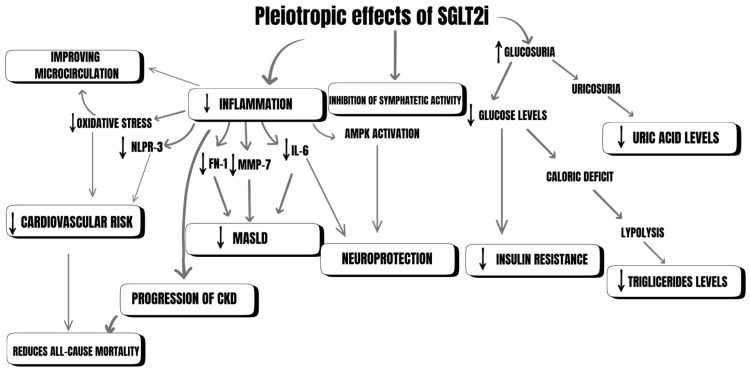
Pleiotropic effects of SGLT2 inhibitors.

Glucose is primarily reabsorbed and filtered in the kidneys through two cotransporters: sodium-glucose cotransporter type 1 (SGLT1) and sodium-glucose cotransporter type 2 (SGLT2). In healthy, normoglycemic adults with a glomerular filtration rate (GFR) of 125 mL/min/1.73 m^2^, approximately 160–180 g of glucose are filtered into the proximal tubule daily. SGLT2 is responsible for about 97% of glucose reabsorption. In the distal tubule of the nephron, SGLT1 is responsible for the reabsorption of approximately 3% of the glucose. The capacity of SGLT1 is largely masked by the function of SGLT2 [8]. In individuals with type 1 and type 2 diabetes, with preserved GFR, around 500–600 g of glucose are filtered into the proximal tubule daily [9]. This is due to hyperglycemia, which leads to a series of hemodynamic and metabolic changes. The cytokines resulting from these changes damage the glomerular filtration barrier, increasing glucose filtration. As the glucose concentration increases, the reabsorption also rises until it reaches approximately 11 mmol/L (198 mg/dL). At this point, the glucose transport system becomes fully saturated, and the maximal reabsorption rate, known as the glucose transport maximum (TmG), is reached. Beyond this threshold, the kidneys can no longer reabsorb glucose, leading to its excretion in the urine, which marks the onset of glycosuria [10]. In healthy adults, TmG is approximately 375 mg/min [8]. TmG increases by about 20% in patients with type 1 diabetes (T1DM) and type 2 diabetes (T2DM) [11,12]. In individuals with type 2 diabetes, there is also overexpression of SGLT2 in the proximal tubule [13]. This is likely a result of the action of angiotensin II (Ang II), hepatocyte nuclear factor 1 alpha (HNF-1α), and tubular growth [14,15]. Combined, the overexpression of SGLT2 with hyperglycemia leads to increased glucose and sodium reabsorption. SGLT2 inhibitors act strongly and selectively on the sodium-glucose cotransporter 2 (SGLT2) located in the proximal tubule of the nephron, inhibiting the reabsorption of glucose and consequently leading to glucosuria [13]. In this way, SGLT2 inhibitors reduce hyperglycemia and its associated negative effects. They also contribute to weight loss, with approximately 300 kcal per day being excreted during SGLT2 inhibitor treatment, leading to a weight loss of about 2–3 kg over several months [16,17]. Although weight loss tends to be more pronounced in clinical trials than in real-world clinical practice. Beyond glycemic control, SGLT2 inhibitors play a crucial role in renal protection. By affecting the MD-Na-K-Cl cotransporter, they reduce proteinuria and hyperfiltration. Their natriuretic properties lead to a decrease in blood pressure, thereby lowering the risk of cardiovascular diseases [18]. Furthermore, these agents influence the renin–angiotensin–aldosterone system (RAAS) by enhancing tubuloglomerular feedback [19,20]. Increased sodium delivery to the macula densa results in afferent arteriole constriction, reducing intraglomerular pressure and hyperfiltration [21]. Although initial natriuresis may activate RAAS, long-term stabilization of this system contributes to sustained nephroprotective effects. Inhibition of SGLT2 plays a crucial role in preventing the progression of kidney damage caused by hyperglycemia and inflammation [22]. Given these benefits, we will further explore the potential additional therapeutic effects of SGLT2 inhibitors and their underlying mechanisms (Figure 1).

### 3.2. Pleiotropic Effects of SGLT-2i

#### 3.2.1. Neuroprotection

Alzheimer’s disease (AD) and Parkinson’s disease (PD) are the two most common neurodegenerative disorders, affecting 39.9 million and 6.2 million people worldwide, respectively. It is estimated that by 2050, the number of people with AD will increase threefold, and by 2040, the number of individuals with PD will rise 2.5 times [23,24]. AD is a progressive and incurable condition that leads to a gradual decline in cognitive function and memory, with its pathophysiology driven by a combination of genetic, environmental, and lifestyle factors. Key mechanisms in AD include the accumulation of amyloid-beta plaques, the formation of neurofibrillary tangles of hyperphosphorylated tau protein, and processes such as oxidative stress, inflammation, and mitochondrial dysfunction [25,26]. On the other hand, PD, also a progressive disorder, primarily affects movement due to the degeneration of dopaminergic neurons in the substantia nigra, causing tremors, rigidity, bradykinesia, and cognitive decline [27]. A key feature of PD is the accumulation of misfolded alpha-synuclein proteins, which form Lewy bodies and contribute to neuronal damage [28]. Both diseases remain incurable, with current treatments focusing on symptom management, while their underlying causes are influenced by a mix of genetic and environmental factors. The pathomechanism of PD is based on a series of changes in brain homeostasis leading to neuroinflammation, which induces the accumulation of alpha-synuclein [29,30]. The mechanisms leading to neuroinflammation include the release of cytokines such as tumor necrosis factor-α (TNF-α)and interleukin-1β (IL-1β) by microglial cells. Additionally, oxidative stress plays a significant role in the pathophysiology of PD [31]. Patients with PD have been found to exhibit reduced activity of key antioxidant enzymes, including superoxide dismutase (SOD), catalase (CAT), and glutathione peroxidase (GPx). Their decreased activity leads to the accumulation of excess reactive oxygen species (ROS), mitochondrial dysfunction, and neuronal damage, particularly affecting dopaminergic neurons in the substantia nigra, which are highly vulnerable to oxidative stress [32,33,34]. Studies show that SGLT2 inhibitors play a significant role in neuroprotection. These compounds are lipid-soluble and achieve a brain-to-serum ratio of 0.3 to 0.5, allowing them to cross the blood–brain barrier and act directly in the central nervous system [35]. A 2023 study examined the effects of empagliflozin on key pathogenic mechanisms of Parkinson’s disease, including, among others, the AMPK/SIRT-1/PGC-1α and Wnt/β-catenin signaling pathways [36]. Disruptions in the AMPK/SIRT-1/PGC-1α pathway lead to a reduction in both the number and function of mitochondria, increasing the vulnerability of dopaminergic neurons to oxidative stress and cell death [37,38]. Similarly, abnormalities in the Wnt/β-catenin pathway can cause impairments in neuronal proliferation, differentiation, and survival, contributing to disease progression [39]. The study demonstrated that empagliflozin can reduce oxidative stress by improving mitochondrial function and decreasing the production of reactive oxygen species (ROS). Additionally, it exhibits anti-inflammatory properties by limiting microglial activation and lowering pro-inflammatory cytokine levels in the brain [40]. Furthermore, empagliflozin activates the AMPK/SIRT-1/PGC-1α pathway, leading to the phosphorylation and activation of PGC-1α, as well as its deacetylation by SIRT-1, thereby enhancing its activity. As a result, mitochondrial biogenesis increases, and neuronal energy metabolism improves. Empagliflozin also modulates the Wnt/β-catenin pathway, supporting neurogenesis, differentiation, and neuronal survival. Another study with empagliflozin shows that it improves neuroplasticity in PD [41]. The increasing number of individuals suffering from metabolic and neurodegenerative diseases worldwide has led to a significant rise in research in this area. Inflammatory processes play a central role in both type 2 diabetes and Alzheimer’s disease [42]. Research has highlighted a connection between these conditions, showing that patients with Type 2 Diabetes Mellitus (T2DM) have a 53% increased relative risk of developing Alzheimer’s disease (AD) compared to individuals without diabetes [43]. In this regard, studies conducted with empagliflozin show that it reduces amyloid plaques in the brains and hippocampus of mice [44]. Additionally, dapagliflozin has shown promising results in preventing both amyloid-beta accumulation and tau phosphorylation in rat models [45]. SGLT2 inhibitors also affect the mTOR pathway, which is known for its role in increasing beta-amyloid levels and tau protein hyperphosphorylation [45]. Furthermore, BDNF is a key factor in neuroplasticity, and it is known that SGLT2 inhibitors can increase its levels [41]. A 2024 cohort study involving 1,348,362 patients with type 2 diabetes who initiated antihyperglycemic treatment (2014–2019), assessed using the Korean National Health Insurance Service database, examined the association between SGLT2 inhibitor use and the incidence of Parkinson’s disease (PD), Alzheimer’s disease (AD), and vascular dementia (VaD) [46]. The analysis revealed that SGLT2i use was associated with a reduced risk of AD (HR 0.81, 95% CI 0.76–0.87), VaD (HR 0.69, 95% CI 0.60–0.78), and PD (HR 0.80, 95% CI 0.69–0.91). These findings remained consistent across various subgroups, including sex, comorbidities, and concurrent medications. This study provides Class II evidence supporting the neuroprotective effects of SGLT2 inhibitors.

#### 3.2.2. Decreasing the Risk of Developing Type 2 Diabetes

**Figure 2:**
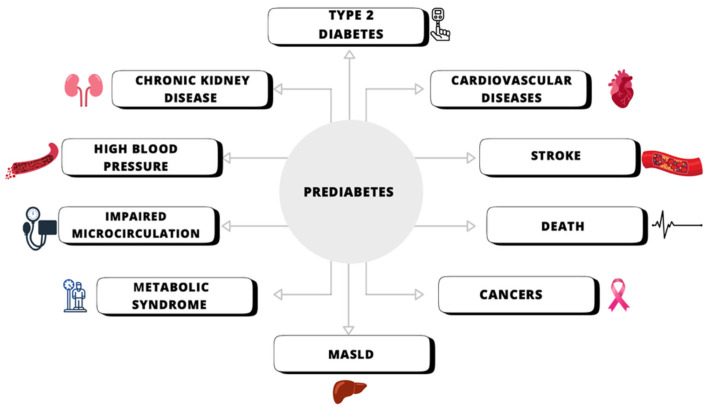
The potential risk associated with prediabetic state.

Prediabetes is an intermediate stage of carbohydrate metabolism disruption between normal fasting glucose levels and glucose tolerance and full-blown diabetes. It can be classified into impaired fasting glucose (IFG) and impaired glucose tolerance (IGT). Among individuals with prediabetes, 5–10% will progress to type 2 diabetes. At the prediabetes stage, typical microvascular changes associated with diabetes may already occur. Currently, both the Polish Diabetes Society and the American Diabetes Association recommend the use of metformin alongside lifestyle modifications for individuals with prediabetes, particularly those with impaired fasting glucose and impaired glucose tolerance, a body mass index (BMI) of ≥35 kg/m^2^, under 60 years of age, and for women who have previously had gestational diabetes. However, it is worth noting that some studies suggest that metformin may not be effective in treating prediabetes [47]. The graphic below illustrates the potential health risks associated with prediabetes (Figure 2).

A 2022 meta-analysis demonstrated that the use of SGLT2 inhibitors is associated with a reduced risk of developing type 2 diabetes in individuals with prediabetes, coronary artery disease, and chronic kidney disease [48]. Although dapagliflozin was associated with a 1.8-fold greater reduction in the risk of new-onset diabetes compared to empagliflozin (32% for dapagliflozin versus 18% for empagliflozin), no statistically significant difference was found between these two classes of SGLT2 inhibitors. Both medications were well tolerated regarding hypoglycemic events, even in individuals with prediabetes, heart failure, or chronic kidney disease. Type 2 diabetes is associated with insulin resistance, dysfunction of beta cells both qualitatively and quantitatively, and their ability to adequately produce insulin. During the progression of the disease, the mass of beta cells in the pancreas changes. It has been discovered that the mass of beta cells can sometimes be reduced even before the diagnosis of type 2 diabetes. Although later on, there is a compensatory increase in beta cell mass in response to growing insulin resistance, this has been observed primarily in the Caucasian population, not in the Japanese populace [49,50]. SGLT2 inhibitors have shown a protective effect on beta cells, positively influencing insulin secretion. This effect may be linked to the anti-inflammatory action of these agents, given that beta cells have limited anti-inflammatory mechanisms and are highly susceptible to damage [51]. Treatment with empagliflozin in patients with type 2 diabetes has also been demonstrated to enhance pancreatic beta cell function, as measured by the insulin secretion to insulin resistance ratio during a hyperglycemic clamp test [52,53].

#### 3.2.3. Managing MASLD

The pathogenesis of MASLD involves hepatic steatosis, inflammation, and fibrosis, often associated with insulin resistance and metabolic syndrome. Metabolic dysfunction-associated steatotic liver disease (MASLD) is commonly associated with type 2 diabetes. According to a meta-analysis published in 2019, approximately 55.5% of individuals with type 2 diabetes worldwide suffer from MASLD [54]. The comorbidity of these two conditions presents a significant clinical problem, as the presence of both pathologies is associated with a greater health risk than either condition alone [55,56,57]. The disease progression starts with simple steatosis (fat accumulation in liver cells) and can advance to more severe stages, such as metabolic dysfunction-associated steatohepatitis (MASH), fibrosis, cirrhosis, and eventually hepatocellular carcinoma [58]. SGLT2 inhibitors appear to exert their effects both directly and indirectly. Glucosuria creates a negative energy balance, which indirectly forces the body to rely on lipids as its primary source of energy. This shift promotes considerable changes, for instance, enhanced fat metabolism, inhibition of leptin, insulin sensitivity, suppression of fibrogenesis and local inflammation. Thus, weight loss and enhanced fatty acid oxidation resulting from SGLT2 inhibitor use may help reduce hepatic fat accumulation in patients with T2DM and MASLD [59]. Empagliflozin was shown to significantly improve liver damage in an animal model of T2DM with MASLD by promoting hepatic macrophage autophagy through the AMPK/mTOR signaling pathway, while also reducing hepatic inflammation [60]. The SGLT2 inhibitor NGI001 was show to inhibit de novo lipogenesis by significantly reducing the expression of FASN and SREBP-1c. It also enhanced fatty acid β-oxidation by increasing the levels of ATGL, CPT1, and PPARα, which consequently reduced fat droplet accumulation in a human fatty liver cell model [61]. Furthermore, a 2018 meta-analysis of randomized controlled trials demonstrated that SGLT2 inhibitors reduced leptin levels, which in turn led to decreased food intake. Additionally, the study found that adiponectin levels, a hormone known to improve insulin sensitivity and possess anti-inflammatory properties, were increased [62]. Inflammation plays a crucial role in the pathogenesis and progression of MASLD [63]. In Hiddo et al., canagliflozins reduced inflammation and fibrosis markers such as IL-6, matrix metallopeptidase 7 (MMP7) and fibronectin 1(FN1) [64]. Additionally, these medications work by suppressing sympathetic nerve activity and enhancing vagal nerve function, which helps reduce inflammation by inhibiting Kupffer cell activation in the liver [65]. Moreover, SGLT2 inhibitors have been shown to activate the AMP-activated protein kinase (AMPK) signaling pathway indirectly by reducing cellular ATP levels. Activation of AMPK enhances fatty acid oxidation and inhibits lipogenesis, contributing to the reduction in hepatic steatosis and inflammation [66]. The overall reduction in free fatty acids is particularly important as it helps prevent hepatic fat accumulation and associated inflammation, which, as mentioned, further progress fibrosis [67]. The clinical trials support this evidence. A 24-week randomized controlled trial (RCT) investigating two groups of patients with T2D and MASLD demonstrated that treatment with dapagliflozin (5 mg/day) led to significant improvements compared to the control group [68]. The study revealed significant decreases in liver steatosis (measured by FibroScan), serum ALT, γ-GT levels, and visceral fat mass. Another retrospective study on 3667 Canadian patients with T2D explored the effects of dapagliflozin, canagliflozin, liraglutide and sitagliptine on ALT levels [69]. ALT levels were lower following treatment with the SGLT2 inhibitors canagliflozin and dapagliflozin compared to liraglutide or sitagliptin (mean follow-up: 4.8 months). Moreover, a sustained and significant reduction in ALT was observed exclusively in the SGLT2 inhibitor group compared to the control. The noteworthy E-LIFT trial revealed that supplementing standard diabetes therapy with 10 mg of empagliflozin for 20 weeks led to a substantial decrease in liver fat, dropping from 16.2% to 11.3%, as assessed by MRI-PDFF [70]. Additionally, an important trial demonstrated that a daily dose of 25 mg empagliflozin significantly reduced liver fat content, as assessed by magnetic resonance spectroscopy [71,72]. Shibuya et al., in a prospective randomized controlled study, showed that lusegliflozin improves liver fat deposition compared to metformin in patients with T2D and MASLD [73]. Similar results were observed in a study with ipragliflozin, which demonstrated comparable effects to pioglitazone and also reduce visceral fat [65].

#### 3.2.4. Reducing Uric Acid

Uric acid is the poorly soluble end product of purine metabolism in humans [74]. Approximately two-thirds of total body urate is produced endogenously, while the remaining one-third is derived from dietary purines [75]. Hyperuricemia can result from increased production or decreased excretion of uric acid. Hyperuricemia is associated with several health conditions, including gout, kidney stones, chronic kidney disease, insulin resistance, T2D, and prediabetes [76,77]. There is a bilateral connection between T2D and hyperuricemia [78]. Patients with T2D are more likely to have higher levels of uric acid, and those with hyperuricemia are at greater risk of developing T2D. Combined, both conditions are associated with increased risk of comorbidity and mortality [79]. SGLT2 inhibitors were reported to lower serum uric acid (SUA) levels. The reduction in uric acid levels seems to occur quickly, with notable decreases seen within the first week of starting treatment [80,81]. The reduction in SUA levels by SGLT2 inhibitors is primarily attributed to their uricosuric effect [82]. The elevated glucose concentration in the tubular lumen competitively inhibits urate reabsorption transporters, such as URAT1 and GLUT9, leading to increased uric acid excretion [83]. This mechanism results in a decrease in serum uric acid levels. Additionally, SGLT2 inhibitors may influence intracellular metabolic pathways that affect uric acid production. By modulating nutrient-sensing pathways, such as AMP-activated protein kinase (AMPK) and sirtuin 1 (SIRT1), SGLT2 inhibitors can reduce the activity of enzymes involved in uric acid synthesis, further contributing to lower SUA levels [84,85]. Furthermore, SGLT2 inhibitors lower uric acid production by inhibiting xanthine oxidase activity through their impact on the RAAS and sympathetic nervous system, while also reducing inflammation [85,86]. Overall studies indicated that SGLT2i, by decreasing SUA, diminishes cardiovascular risk, hospitalization for heart failure, and slows progression of chronic kidney disease [87,88].

#### 3.2.5. SGLT2 Inhibitors and Anemia

It has also been observed that SGLT2 inhibitors influence the state of red blood cell parameters. Initially, during treatment with SGLT2 inhibitors, an increase in hematocrit and hemoglobin levels is noted, which may be associated with hemoconcentration due to natriuresis and plasma volume contraction, both linked to their mechanism of action [89]. However, beyond this, SGLT2 inhibitors have been found to stimulate erythropoiesis through the activation of hypoxia-inducible factor-2 alpha (HIF-2α), which induces the production of erythropoietin (EPO) [90]. The exact mechanism remains unclear, though two theories have been proposed. The first suggests that SGLT2 inhibitors increase sodium delivery to the distal nephron segments, leading to enhanced metabolism and oxygen consumption by tubular cells. This results in local hypoxia, which stimulates interstitial fibroblasts to produce EPO. The second theory proposes that SGLT2 inhibitors activate an alternative metabolic pathway for EPO synthesis in the liver. This is particularly relevant in chronic kidney disease (CKD), where the kidneys progressively lose their ability to synthesize EPO. SGLT2 inhibitors may activate hepatic sirtuin-1 (SIRT-1), which in turn stimulates HIF-2α, leading to increased EPO production independently of hypoxia. Drugs commonly used to treat anemia in CKD, such as HIF-PHD inhibitors or erythropoiesis-stimulating agents (ESAs), require hypoxia to function. HIF-PHD inhibitors activate both HIF-2α and HIF-1α, the latter of which has been associated with increased cardiac fibrosis and destabilization of atherosclerotic plaques. In contrast, SGLT2 inhibitors selectively stimulate HIF-2α while inhibiting HIF-1α, which may underlie their cardioprotective effects. Thus, unlike HIF-PHD inhibitors or ESAs, SGLT2 inhibitors do not increase the risk of cardiovascular complications but rather appear to reduce it [91]. SGLT2 inhibitors contribute to increased hemoglobin levels, thereby supporting anemia management [92,93]. Additionally, by suppressing pro-inflammatory signaling pathways and enhancing nutrient deprivation signals, SGLT2 inhibition lowers both hepcidin and ferritin levels. As a result, it promotes the absorption of iron from the gastrointestinal tract and the mobilization of iron from the reticuloendothelial system (due to decreased hepcidin) and from intracellular stores (as ferritin levels decrease) [94]. A 2024 cohort study investigated the effects of sotagliflozin—a dual SGLT1 and SGLT2 inhibitor—on hemoglobin levels in patients with type 2 diabetes at stages 3 and 4 of CKD [89]. Over 26 weeks, an increase in hemoglobin concentration was observed: 0.39 g/dL (200 mg; 95% CI 0.21–0.56) and 0.41 g/dL (400 mg; 95% CI 0.24–0.59) versus placebo (*p* < 0.0001). This suggests that sotagliflozin increased the likelihood of anemia resolution. Among participants who were not anemic but at risk of developing anemia, the use of sotagliflozin demonstrated a reduction in the risk of onset, although this finding was not statistically significant. Furthermore, in a post hoc analysis of the CREDENCE trial, the effects of canagliflozin versus placebo on haemoglobin and haematocrit in patients with type 2 diabetes and chronic kidney disease were evaluated. The results showed that canagliflozin significantly increased haemoglobin and haematocrit levels and reduced the risk of anaemia-related events, including the need for erythropoiesis-stimulating agents, compared to placebo [95]. Similar results have been observed in such trials as DAPA-HF (dapagliflozin), EMPEROR-Reduced (empagliflozin) or DAPA-CKD (dapagliflozin) [92,96,97].

#### 3.2.6. Reducing Inflammation

Chronic low-grade inflammation plays a significant role in the pathogenesis of most diseases, especially metabolic disorders. Some of the anti-inflammatory mechanisms of action of SGLT2 inhibitors have already been discussed in this study. These agents act on multiple levels in the progression of inflammation and have shown anti-inflammatory benefits in both diabetic and non-diabetic conditions [98,99]. The anti-inflammatory effects of SGLT2 inhibitors are thought to involve modulation of key signaling pathways, including nuclear factor kappa B (NF-κB), mitogen-activated protein kinase (MAPK), and Janus kinase/signal transducer and activator of transcription (JAK/STAT). The suppression of the NF-κB signaling pathway, a key regulator of inflammation, significantly reduces the activation of the Toll-like receptor 4 (TLR4)/NF-κB pathway in macrophages, which leads to decreased production of inflammatory molecules such as IL-6 and TNF-α [100]. Moreover, in macrophages, empagliflozin has been shown to block both the MKK4/7-JNK pathway (part of MAPK signaling) and the JAK2-STAT1/3 pathway, leading to reduced production of pro-inflammatory molecules [101]. In vitro studies have demonstrated that empagliflozin also attenuates the release of pro-inflammatory cytokines and mediators by downregulating these pathways, irrespective of glucose concentration [102]. Additionally, SGLT2 inhibitors may influence nutrient-sensing pathways, such as adenosine monophosphate-activated protein kinase (AMPK) and mammalian target of rapamycin (mTOR), which play roles in cellular metabolism and inflammation [103]. In type 2 diabetes, these pathways are impaired, resulting in a notable decrease in the regulatory factors that usually preserve cellular homeostasis [104]. At the molecular level, SGLT2 inhibitors can directly interact with and inhibit glucose transporters GLUT1 and GLUT4 in cardiac myocytes, thereby decreasing glucose uptake into the cells. This reduction in intracellular glucose levels subsequently triggers the activation of AMPK. The activation of AMPK effectively inhibits mTOR, especially through its mTORC1 [105,106]. In summary, by modulating these pathways, SGLT2 inhibitors can reduce inflammatory responses associated with metabolic stress. Additionally, the NLRP3 inflammasome plays a key role in mediating inflammation in the kidney and heart, with its activation linked to the progression of diabetic kidney disease and the development of atherosclerosis and heart failure. SGLT-2 inhibitors have been shown to attenuate NLRP3 activation, contributing to an anti-inflammatory environment [107]. As it has been previously mentioned, SGLT2 inhibitors can influence macrophages. Macrophages exhibit plasticity, primarily existing in two distinct phenotypes [108]. M1 macrophages, activated by Th1 cytokines or bacterial lipopolysaccharides (LPS), produce pro-inflammatory cytokines and play a key role in immune defense. Contrarily, M2 macrophages, activated by Th2 cytokines, exert anti-inflammatory effects by releasing cytokines such as IL-1 receptor antagonist and IL-10. The balance between M1 and M2 macrophages is crucial for inflammation regulation and tissue repair [109]. M1 macrophages dominate during acute inflammation to eliminate pathogens, but prolonged activation can lead to tissue damage. To mitigate this, M2 macrophages release anti-inflammatory mediators that suppress inflammation and support tissue regeneration. Research consistently shows that SGLT2 inhibitors facilitate the shift from M1 to M2 macrophages, promoting an anti-inflammatory environment [108,110,111]. This effect has been observed across various SGLT2 inhibitors and disease models. It is worth mentioning that flozins, by reducing weight, also decrease adipose tissue mass, which is known to have a significant impact on inflammatory response [112]. The visceral white adipose tissue(WAT)is a source of pro-inflammatory cytokines(adipokines) [113,114]. Several clinical studies utilizing advanced imaging techniques have shown that SGLT2 inhibitors effectively decrease visceral adipose tissue. Additionally, bioimpedance spectroscopy research has confirmed that SGLT2 inhibitors reduce both visceral and subcutaneous fat while maintaining lean body mass [115].

In summary, multiple studies have confirmed that this multi-pathway modulation by SGLT2 inhibitors results in decreased production of key inflammatory mediators, including TNF-α, IL-1β, IL-6, and IFN-γ, as well as various pro-inflammatory chemokines.

#### 3.2.7. Restoring Circadian Metabolic Rhythms and Enhancing Catabolic Processes in Type 2 Diabetes

SGLT2 inhibitors promoted a catabolic metabolic shift via sustained overnight glucosuria, altering fuel utilization and enhancing autophagy. By increasing the glucagon-to-insulin ratio, they depleted hepatic glycogen and activated gluconeogenesis, primarily using circulating amino acids. This was accompanied by a fuel switch from glucose to free fatty acids and a shift in mitochondrial morphology from fission to fusion. As amino acids and insulin levels declined, mTORC1 was inhibited, promoting the clearance of dysfunctional organelles. Upon resumption of feeding, anabolic pathways were reactivated, facilitating organelle and protein regeneration. This restoration of diurnal metabolic rhythms may extend benefits beyond glycemic control, with potential therapeutic implications in non-diabetic diseases [116].

#### 3.2.8. SGLT2 Inhibitors and PCOS

Polycystic ovary syndrome (PCOS) is an endocrine disorder affecting 4% to 20% of the worldwide population [117]. The pathophysiological mechanisms contributing to this syndrome include hyperandrogenism, follicular dysfunction, insulin resistance, and inflammation. However, the complete pathogenesis of PCOS remains not fully understood. Due to these disruptions in the body’s homeostasis, patients are at a higher risk of infertility, endometrial cancer, metabolic syndrome, cardiovascular diseases, and their associated complications. Currently, treatment is mainly symptomatic, with metformin being proposed as a drug to regulate insulin secretion disturbances. Thus, there is a growing need for a new, comprehensive approach to PCOS treatment. There were already a considerable number of trials concerning treating patients with PCOS with SGLT-2i, where they explored the potential effect on both metabolic parameters, such as insulin resistance(IR), weight and hormone serum levels. From a hormonal perspective, the results are mixed, with some studies indicating a significant reduction in the free androgen index; in others, there was no significant reduction [118,119,120]. It is worth mentioning that the best results in these studies were achieved in groups receiving a combination of SGLT2 inhibitors and metformin. In three of the four studies that assessed dehydroepiandrosterone sulfate (DHEAS), a reduction in levels was observed [119,120,121,122]. Androstendione levels were decreased in these studies [118,119,120]. Two of five studies showed a significant increase in SHBG levels. Empagliflozin and dapagliflozin increased SHBG, while canagliflozin and metformin had no significant effect [118,119,120,122]. The cardiovascular benefits of SGLT2 inhibitors are especially important for PCOS patients, who are at higher cardiovascular risk. Research indicates that these drugs can lower blood pressure and enhance cardiovascular health [123,124]. SGLT2i have been shown to significantly improve body weight, BMI, and overall body composition [125,126]. Short-term studies with dapagliflozin have reported substantial enhancements in blood glucose control and insulin resistance, as indicated by HOMA-IR [126,127]. Table 1 summarizes selected studies investigating the effects of SGLT2 inhibitors on hormonal and metabolic outcomes in PCOS.

The table summarizes selected experimental, meta-analytical, and clinical trial data investigating the effects of SGLT2 inhibitors in the management of PCOS, with a focus on metabolic, hormonal, and anthropometric outcomes.

#### 3.2.9. Klotho Protein and Its Modulation by SGLT2 Inhibitors

Klotho is a membrane-bound and soluble protein primarily expressed in the distal renal tubules, known for its anti-aging, antioxidative, and anti-fibrotic properties. It regulates phosphate and calcium homeostasis, inhibits insulin/IGF-1 signaling, and reduces inflammation and oxidative stress—key contributors to the pathogenesis of diabetic kidney disease (DKD) and cardiovascular dysfunction [129,130]. In diabetes, Klotho levels decline significantly, contributing to the progression of renal fibrosis, endothelial dysfunction, and accelerated vascular aging [129,131]. Therefore, restoring Klotho expression is gaining attention as a promising therapeutic approach to prevent or delay cardiorenal complications. SGLT2 inhibitors (SGLT2i), originally developed for blood glucose control, have demonstrated significant pleiotropic effects. One emerging mechanism underlying their reno- and cardioprotective effects is the modulation of Klotho protein expression. In a clinical study by Mora-Fernández et al., treatment with empagliflozin, canagliflozin, or dapagliflozin significantly increased urinary and serum Klotho concentrations in patients with early DKD—up to a 39% increase in urinary levels—independent of glycemic changes [129]. Similarly, Topchii et al. reported a 23% increase in serum Klotho after six months of dapagliflozin therapy in diabetic nephropathy patients, supporting a systemic benefit beyond glucose lowering [131]. Mechanistically, SGLT2 inhibitors enhance Klotho expression both at the transcriptional level and via post-translational pathways. Wolf et al. demonstrated in vitro that empagliflozin and canagliflozin upregulated Klotho expression in renal proximal tubular cells (HK-2 and MDCK lines) and promoted its shedding via ADAM17, resulting in increased soluble Klotho in the extracellular environment [132]. In animal models, Abbás et al. found that empagliflozin enhanced renal Klotho expression and attenuated interstitial fibrosis and inflammation in a rat model of unilateral ureteral obstruction (UUO) [130]. Similar protective findings were observed by Castoldi et al., where SGLT2 inhibition prevented renal fibrosis in a cyclosporine nephropathy model, likely through a Klotho-dependent mechanism [133]. From a vascular perspective, elevation of soluble Klotho is also associated with improved endothelial function and reduced arterial stiffness. Karalliedde et al. showed that dapagliflozin increased soluble Klotho levels and improved arterial compliance, indicating a vasculoprotective role mediated at least in part by this protein [134]. Several reviews have summarized the emerging evidence that SGLT2 inhibitors modulate Klotho as a potential mechanism for their broad protective effects. Wolf et al. described Klotho upregulation as a downstream effect of altered cellular energy and redox status induced by SGLT2 inhibition [132]. Additional work by the Frontiers in Endocrinology editorial group highlighted that Klotho restoration by SGLT2 inhibitors may counteract oxidative stress and fibrosis in DKD [135]. Broader analyses, including those by Chen et al. and others, propose Klotho as a key anti-fibrotic and anti-inflammatory mediator that may explain long-term organ protection with SGLT2i [136,137]. In summary, Klotho is a critical factor in renal and vascular homeostasis, and its suppression in diabetes contributes significantly to DKD progression and cardiovascular deterioration. SGLT2 inhibitors restore Klotho expression through diverse mechanisms, representing an important part of their therapeutic profile. Understanding and leveraging this pathway may enhance future treatment strategies in patients with diabetes and chronic kidney disease.

#### 3.2.10. Potential Role of SGLT2 Inhibitors in Oncology

Initially developed for type 2 diabetes, SGLT2 inhibitors (SGLT2i) have shown promising anticancer activity through inhibition of tumor metabolism, induction of apoptosis, immune modulation, and disruption of oncogenic signaling.

##### Disrupting Tumor Glucose Metabolism and Cell Growth

Overexpression of SGLT2 in cancers such as pancreatic, thyroid, colorectal, and NSCLC makes it a viable therapeutic target. SGLT2i (e.g., canagliflozin) reduces glucose uptake and activates AMPK, thereby suppressing AKT/mTOR signaling, leading to cell cycle arrest and apoptosis in thyroid cancer cells [138,139]. In colorectal cancer, they induce mitochondrial dysfunction and ER autophagy, while in breast cancer, they inhibit lipid metabolism and inflammatory signaling via AMPK [140,141].

##### Antitumor Activity in Animal Models

Animal studies demonstrate broad antitumor efficacy across models of NSCLC, glioblastoma, osteosarcoma, and others [142,143]. Canagliflozin inhibited glioblastoma by activating AMPK and suppressing glycolysis. In osteosarcoma, SGLT2i enhanced CD8^+^/CD4^+^ T cell infiltration via STING/IRF3 activation [143,144].

##### Modulation of Immune Responses and Tumor Microenvironment

SGLT2i can augment immune responses by promoting PD-L1 degradation in NSCLC and ovarian cancer cells, enhancing cytotoxic T cell activity [145]. STING/IRF3 pathway activation further supports immune-mediated tumor suppression, suggesting potential synergy with checkpoint inhibitors [144].

##### Emerging Clinical Evidence

Preliminary clinical findings are encouraging. A Taiwanese cohort study showed reduced cancer-specific and all-cause mortality in diabetic cancer patients using SGLT2i [146]. A phase 1b trial of dapagliflozin with chemotherapy in pancreatic cancer showed safety and early efficacy [147], while meta-analyses report reduced cancer mortality, especially in breast cancer [148].

##### Molecular Mechanisms and Therapeutic Perspectives

SGLT2i targets cancer metabolism by inhibiting glycolysis, disrupting mitochondrial function, and modulating key pathways such as AMPK/mTOR, Hippo/YAP, and STING/IRF3, leading to apoptosis and enhanced immune activity [138,139,144,149]. Future directions include identifying SGLT2-high tumors and developing combination therapies.

#### 3.2.11. Emerging Role of SGLT2 Inhibitors in Atrial Fibrillation Prevention

Atrial fibrillation (AF) is a common arrhythmia associated with significant morbidity and mortality, particularly in patients with metabolic and cardiovascular diseases—populations frequently treated with SGLT2 inhibitors. Emerging evidence suggests that beyond their established benefits in heart failure and diabetes, SGLT2 inhibitors may reduce the incidence of AF. This effect is likely mediated through their anti-inflammatory, antifibrotic, and metabolic actions, which improve cardiac structure and function. Recent clinical studies indicate a lower risk of new-onset AF among patients receiving SGLT2 inhibitors compared to other glucose-lowering therapies [150]. These agents appear to attenuate systemic and myocardial inflammation, reduce oxidative stress, and modulate autonomic nervous system activity, all contributing to arrhythmogenesis. Furthermore, SGLT2 inhibitors improve cardiac metabolism and reduce left atrial remodeling—key factors implicated in AF development. A 2022 meta-analysis supports these findings, showing a significant reduction in AF incidence with SGLT2 inhibitor therapy in patients with type 2 diabetes and heart failure [151]. Additional studies reveal benefits in non-diabetic populations, suggesting a broader cardioprotective role [152]. Emerging randomized controlled trials also highlight potential decreases in AF-related hospitalizations and adverse cardiovascular outcomes [153]. Despite promising data, the precise mechanisms underlying the antiarrhythmic effects of SGLT2 inhibitors require further elucidation. Dedicated prospective trials focusing on AF prevention are necessary to confirm these benefits and optimize treatment strategies in high-risk patients. Table 2 contains the key references cited throughout the entire manuscript and highlights the pleiotropic mechanisms through which SGLT2 inhibitors exert their effects in specific pathways.

## 4. Conclusions

SGLT2 inhibitors have evolved from glucose-lowering agents into multifunctional drugs with wide-ranging effects on metabolic and systemic diseases. Their pleiotropic actions—including neuroprotection by reducing oxidative stress and inflammation, anti-inflammatory effects, organ protection, improvements in liver health, anemia correction, and uric acid reduction—highlight their expanding therapeutic potential beyond diabetes. Moreover, emerging evidence suggests benefits in restoring circadian metabolic rhythms and managing endocrine disorders such as polycystic ovary syndrome.

Despite promising preclinical data and early clinical findings, much remains to be established regarding the clinical translation of these effects. Future research should focus on large-scale, long-term clinical trials to assess safety, optimal dosing, and efficacy, particularly in non-diabetic populations and patients with comorbidities. Understanding the mechanisms underlying these diverse actions will be crucial to optimizing therapeutic use and tailoring treatment to specific patient groups.

Priority areas include investigating neurodegenerative diseases, chronic kidney and liver conditions, reproductive endocrinology, and oncology, where early results are encouraging but preliminary. Bridging the translational gap requires integrating mechanistic insights with clinical evidence, alongside real-world studies to guide precision medicine approaches. Overall, ongoing and future research will be essential to fully harness the broad therapeutic benefits of SGLT2 inhibitors, extending their impact far beyond glycemic control.

## Figures and Tables

**Table 1:** Clinical and preclinical studies evaluating the effects of SGLT2 inhibitors in women with polycystic ovary syndrome (PCOS).

Author	SGLT2i	Study Design	Outcomes
**Javed et al., 2019** [119]	Empagliflozin	Randomized controlled trial, comparing effects of empagliflozin vs. metformin on women with PCOS.	Empagliflozin was more effective than metformin in reducing weight, body fat, and waist/hip circumference.
**Sinha et al., 2022** [127]	N/A	Meta-analysis of prospective trials comparing SGLT-2i group to control group.	Reduction in body weight, fasting plasma glucose, insulin resistance, improvement in DHEAS levels.
**Khan et al., 2021** [128]	Canagliflozin	Animal study with Sprague Dawley rats, divided into six groups, tested with canagliflozin and metformin	Canagliflozin alone and in combination shows significant hormonal improvements compared to placebo; beter cycle regularization with combination

**Table 2:** Summary of SGLT2inhibitors’ pleiotropic effects.

Therapeutic Area	Proposed Mechanism(s)	Key Reference
Atrial Fibrillation Prevention	Reduced atrial fibrosis, improved mitochondrial function, ionic balance, and anti-inflammatory effects	[150,151]
Oncology	Inhibition of glycolysis, AMPK/mTOR modulation, autophagy induction, and immune checkpoint regulation	[138,139,140,141]
PCOS	Improved insulin sensitivity, reduced hyperandrogenism and weight loss	[127,128]
Circadian Rhythm Restoration	Glucosuria-induced catabolism, mTORC1 inhibition, autophagy activation, and metabolic rhythm normalization	[116]
Klotho Modulation	Upregulation of Klotho expression, oxidative stress reduction	[129,130]
Anti-inflammatory Effects	NLRP3 inflammasome inhibition, cytokine suppression, and AMPK pathway activation	[98,100]
Anemia Management	Increased erythropoietin production, improved renal oxygenation, and reduced hepcidin	[89,90]
Uric Acid Reduction	Uricosuric effect via inhibition of renal tubular urate reabsorption	[80,81]
MASLD Management	Improved hepatic insulin sensitivity, reduced steatosis, and anti-inflammatory actions	[60,61]
T2D Prevention	Enhanced glucose control, reduced insulin resistance,	[49]
Neuroprotection	Reduction in stress and inflammation, improved mitochondrial function, modulation of AMPK/SIRT1/PGC-1α and Wnt/β-catenin pathways.	[35,37,38]

## Data Availability

Publicly available datasets were analyzed in this study.
